# Temperature stress and disease drives the extirpation of the threatened pillar coral, *Dendrogyra cylindrus*, in southeast Florida

**DOI:** 10.1038/s41598-021-93111-0

**Published:** 2021-07-08

**Authors:** Nicholas P. Jones, Lystina Kabay, Kathleen Semon Lunz, David S. Gilliam

**Affiliations:** 1 Nova Southeastern University, Halmos College of Arts and Sciences, Dania Beach, FL USA; 2 National Operations Center, Bureau of Land Management, Denver, CO USA

**Keywords:** Marine biology, Ecology, Climate-change ecology, Population dynamics, Biodiversity, Conservation biology

## Abstract

Rare species population dynamics can elucidate the resilience of an ecosystem. On coral reefs, climate change and local anthropogenic stressors are threatening stony coral persistence, increasing the need to assess vulnerable species locally. Here, we monitored the threatened pillar coral, *Dendrogyra cylindrus*, population in southeast Florida, USA, in relation to consecutive heat stress events in 2014 and 2015. In the fall of each year, *D. cylindrus* colonies bleached following intense thermal stress and by June 2020 all monitored colonies died from a white-syndrome type disease. This resulted in the ecological extinction of *D. cylindrus* in the Southeast Florida Coral Reef Ecosystem Conservation Area (ECA). White-syndrome type disease was first seen in February 2014 on four colonies (19% prevalence) near the major international port, Port Everglades and disease prevalence peaked in fall 2015 (58%). Disease prevalence increased with maximum water temperature, while disease related mortality increased with mean water temperature. Our findings suggest that thermal stress exacerbated underlying stony coral disease, resulting in an outbreak contributing to the ecological extirpation of *D. cylindrus* in the ECA. We suggest that stony coral resilience is severely compromised by chronic environmental disturbance which hinders community recovery.

## Introduction

Rare species are predicted to be increasingly vulnerable to extinction under climate change and growing anthropogenic stress^1–4^. Species rarity may result from small geographic range, narrow habitat tolerance or low population size^5^. While species can persist in low abundance^6,7^, environmental change may cause a population to become fragmented or reduce intrinsic resistance to stress, leading to extirpation^1^. Consequently, rare species not only create diversity, but can be key in detecting underlying ecological change^8^ with studies increasingly finding they contribute disproportionately to functional richness, specialization and originality of an assemblage^9–11^. In coral reef ecosystems, diversity is positively related to ecosystem function, subsequently promoting resilience^12,13^. The degradation of many coral reefs in the Anthropocene has increased the need to assess ecosystem health and resilience^14^. Monitoring the population dynamics of rare species is an integral part of these assessments.

In recent decades, environmental stress has increased on coral reefs, compromising the health of stony corals and contributing to population decline^15,16^. Thermal stress^17,18^, elevated nutrient pollution^19,20^ and sedimentation^21^ have all been linked to coral bleaching, disease and mortality. Monitoring conducted on broad spatial and temporal scales can capture largescale stony coral community changes in relation to environmental stressors^22,23^; however rare species are rarely captured in these studies. With heightened risk of extinction under climate change and increasing anthropogenic pressure, it is critical to assess threatened, rare species population dynamics through targeted assessments.

A conspicuous species on Caribbean coral reefs, the pillar coral *Dendrogyra cylindrus* is primarily found in low abundance throughout its geographical range^24–26^. Following biological review performed by National Oceanic and Atmospheric Administration-National Marine Fisheries Service (NOAA-NMFS)^27^, *D. cylindrus* met the criteria as Threatened under the United States Endangered Species Act and was listed in 2014^28^ and as a locally Threatened species under the state of Florida Endangered and Threatened Species Act^29^. It is also classified as Vulnerable under the IUCN Red List of threatened species^30^. Not only is *D. cylindrus* threatened, but it is the only species in its genus and colonies have a columnar growth form uncommon to other coral species in the Caribbean, which likely provides a unique functional trait in southeast Florida. Furthermore, recent evidence suggests steady population decline in parts of its range^31^, which combined with low population density limiting sexual reproduction, suggests an Allee effect in *D. cylindrus*^32^.

**Figure 1:**
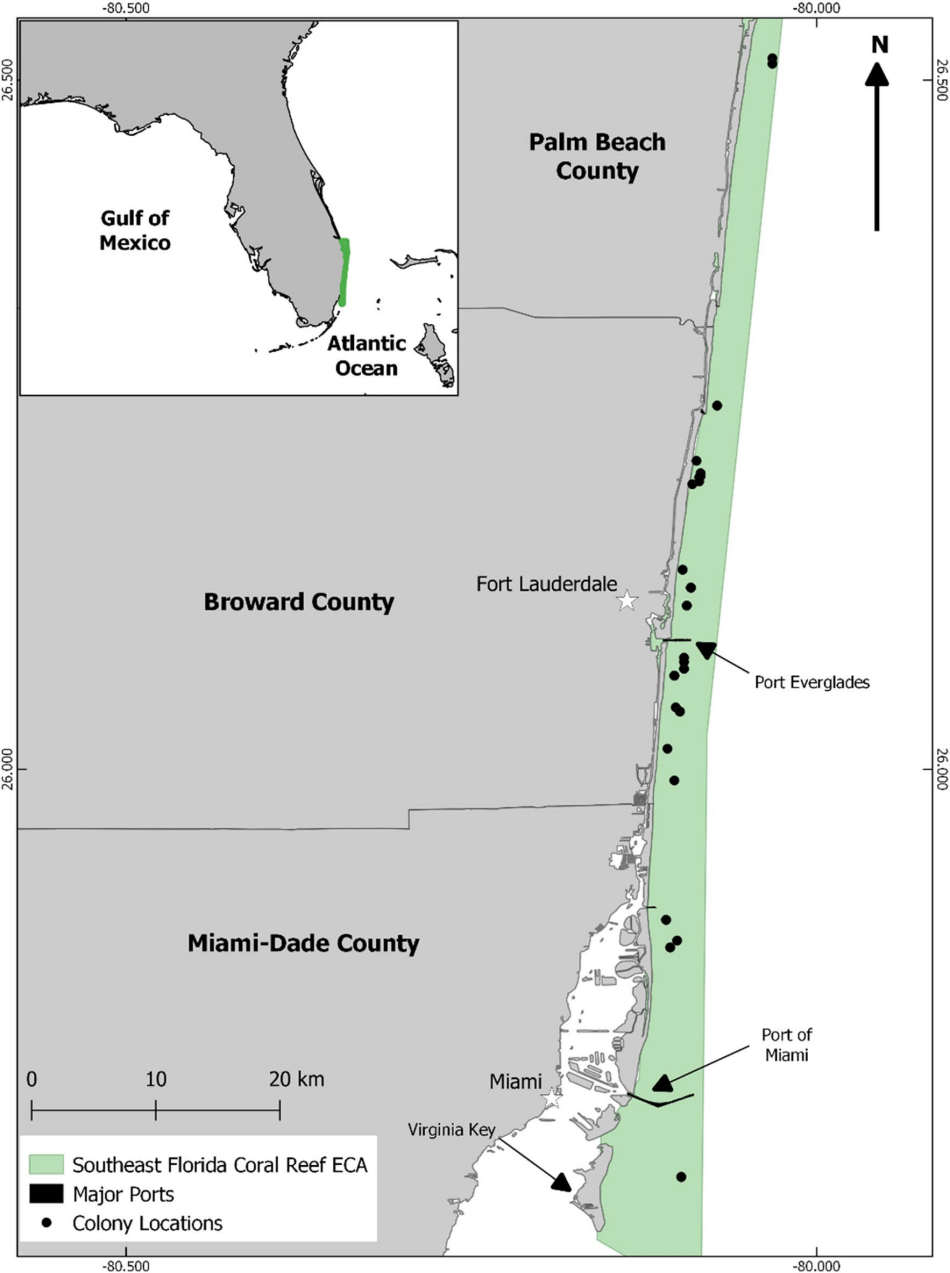
Southeast Florida, with *Dendrogyra cylindrus* colony locations, major ports and specific locations noted. Inset: Florida, with the Southeast Florida Coral Reef Ecosystem Conservation Area highlighted. Figure created using QGIS software 3.18.12.

The northern portion of Florida’s Coral Reef within the Southeast Florida Coral Reef Ecosystem Conservation Area (ECA) lies offshore of a highly urbanized coastline and is the northern limit of *D. cylindrus*’ range. Multiple ports, inlets and sewage outfalls are found along the coastline, contributing to elevated nutrients and contaminants^33^. Two large international ports, Port of Miami and Port Everglades, each move approximately 6.8 million tons (over 1 million 20-foot equivalent units, TEUs) of containerized cargo annually^34,35^. Additionally, six ocean outfalls serving a coastal population of over 6 million discharge partially treated wastewater from 1.5 to four kilometers offshore at approximately 30 m deep^36^. Combined, these have a major influence on the water quality in the ECA^37,38^. From 2014 to 2016, thermal stress and an associated severe stony coral disease outbreak, termed Stony Coral Tissue Loss Disease (SCTLD), reduced stony coral cover in the ECA by 43% and density by 30%^23,39^. A white tissue loss disease of unknown etiology^40^, SCTLD affects over 20 coral species and is considered to be highly virulent in *D. cylindrus*^41^. Outbreak levels (> 5% prevalence) of SCTLD were first reported in the southern end of the ECA off Virginia Key, Miami-Dade County Florida (Fig. 1) following the first of two consecutive bleaching events in September 2014^42^, before spreading north in the ECA and south along Florida’s Coral Reef. In this study, monitoring of *D. cylindrus* colonies in the ECA began in February 2014 and concluded in June 2020, capturing most of the disease outbreak.

This study assessed the status and population dynamics of the threatened pillar coral, *Dendrogyra cylindrus*, in the ECA from 2014 to 2020. The *D. cylindrus* population has not previously been monitored in the ECA and long-term coral reef monitoring efforts in the region have not captured any colonies^43^. Initially, our objective was to conduct the first population assessment of this threatened species in the ECA. Monitoring efforts coincidentally documented disease-related mortality during the same period as a disease outbreak affected many stony coral species in the ECA. As a result, this study ultimately documents the ecological extirpation of *D. cylindrus* at the northern limit of its range and assesses the potential drivers of mortality. We also present new evidence for potential disease outbreak timing and progression in the ECA, which will aid in understanding this unprecedented disease outbreak impacting much of the Caribbean stony coral community.

## Results

### Bleaching, disease and temperature

Thirty-four *D. cylindrus* colonies were visited tri-annually at 12 sites from May 2014 to March 2018. Eighteen of these were also visited in February 2014. Thirty-two additional colonies were monitored annually during this period, three of which were visited in February 2014. *Dendrogyra cylindrus* colonies were found at depths between 4 and 20 m and a mean depth of 6.7 m ± 2.4 m (1 SD). Colonies ranged from 15 to 569 cm maximum diameter with mean diameter 131.5 cm ± 56.4 cm (1 SD; tri-annual mean = 123.1 cm ± 121.2 cm, 1 SD), median diameter 84.5 cm (tri-annual = 82.5 cm) and mean height 69.9 cm ± 56.4 cm (1 SD; tri-annual = 68.7 cm ± 56.5 cm, 1 SD). Forty-seven colonies had an encrusting with pillar growth form, seven had a pillar growth form, four an encrusting growth form and eight were attached fragments. Of the 66 total monitored colonies, 64 (97%) were dead by February 2017, with the last remaining colony dying in June 2020. One colony with less than 5% live tissue was discovered in summer 2020, approximately half a kilometer south of the southernmost monitored colony (B. Walker, Pers. Comms, February 10, 2021).

**Figure 2:**
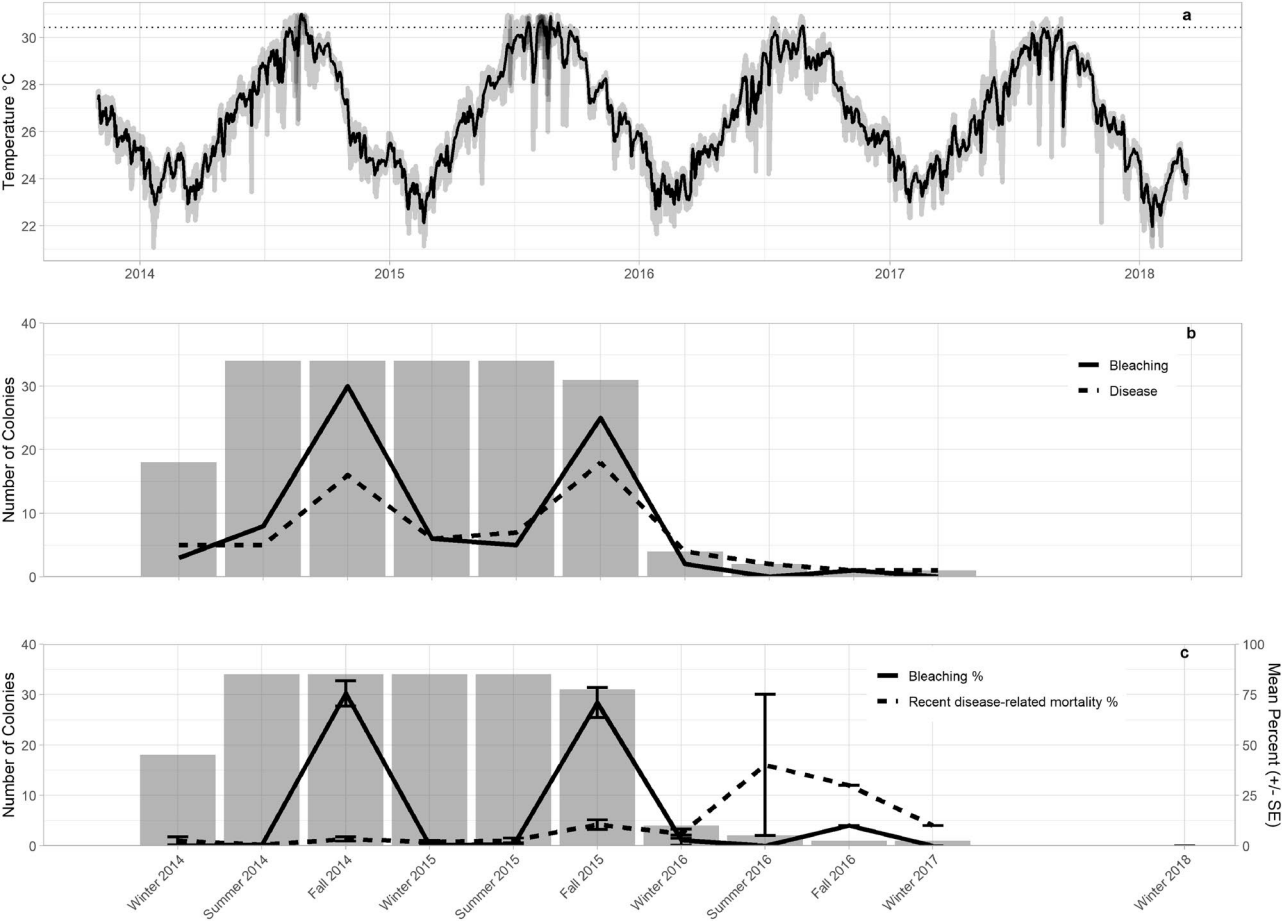
Disease, bleaching and mortality of Dendrogyra cylindrus colonies in relation to water temperature. Top – bottom; (**a**) In situ water temperature (°C), solid black line denotes mean temperature, grey lines reflect temperature from individual sites. Black dotted line = maximum mean monthly temperature during study period (30.41 °C). (**b**) Number of colonies, bleaching and disease over time. Bars = number of live colonies surveyed during monitoring period, black solid line = bleaching prevalence (number of colonies bleached) during monitoring period, black dashed lines = disease prevalence (number of colonies diseased). (**c**) Mean % bleaching and mortality in each monitoring period. Bars = number of live colonies surveyed during monitoring period, black solid line = mean bleaching percentage of life tissue area of those colonies (± SE), black dashed lines = mean disease-related recent mortality (± SE).

Bleaching and disease were recorded on two-thirds of colonies (44 of 66 total colonies) during the study (Fig. 2B,C). The first incidence of white-syndrome type disease-related mortality (8.8% mean recent disease-related colony mortality) was reported on four of 21 colonies monitored in February 2014. Partial bleaching (1–2% of colony live tissue area) was reported on three colonies in February 2014. Bleaching prevalence peaked in fall 2014 (88% of colonies) with mean bleaching percent also peaking in 2014 (75.5 ± 6.4% (± SE)). Bleaching subsided during winter 2015 before 81% of colonies bleached again in fall 2015. Mean bleaching percent was 71 ± 7.4% (± SE) in fall 2015. Disease prevalence peaked in fall 2015 with disease-related mortality observed on 58% of colonies and mean disease-related colony partial mortality of 10.5 ± 2.3% (± SE). Between the fall 2015 and winter 2016 monitoring periods (September 2015–April 2016), disease-related mortality increasingly progressed and 87% of all monitored colonies died. The four surviving tri-annually monitored colonies were observed diseased and remained diseased until they too suffered complete mortality.

Water temperatures exceeded the maximum mean monthly temperature, 30.41 °C, every year (Fig. 2A). Water temperature peaked in summer 2014, with a maximum recorded temperature of 31.86 °C at a Miami-Dade nearshore Southeast Florida Coral Reef Evaluation and Monitoring Project (SECREMP) site. Mean water temperature between monitoring periods peaked in summer 2015, June–September, with a mean temperature of 30.22 °C at a Miami-Dade County nearshore SECREMP site. Disease prevalence was positively related to maximum water temperature (GLM, p = 0.01), while recent disease-related mortality was positively correlated with mean water temperature between monitoring periods (Spearman’s rank correlation, p < 0.0001, rho = 0.34).

### Spatial pattern of disease

**Figure 3:**
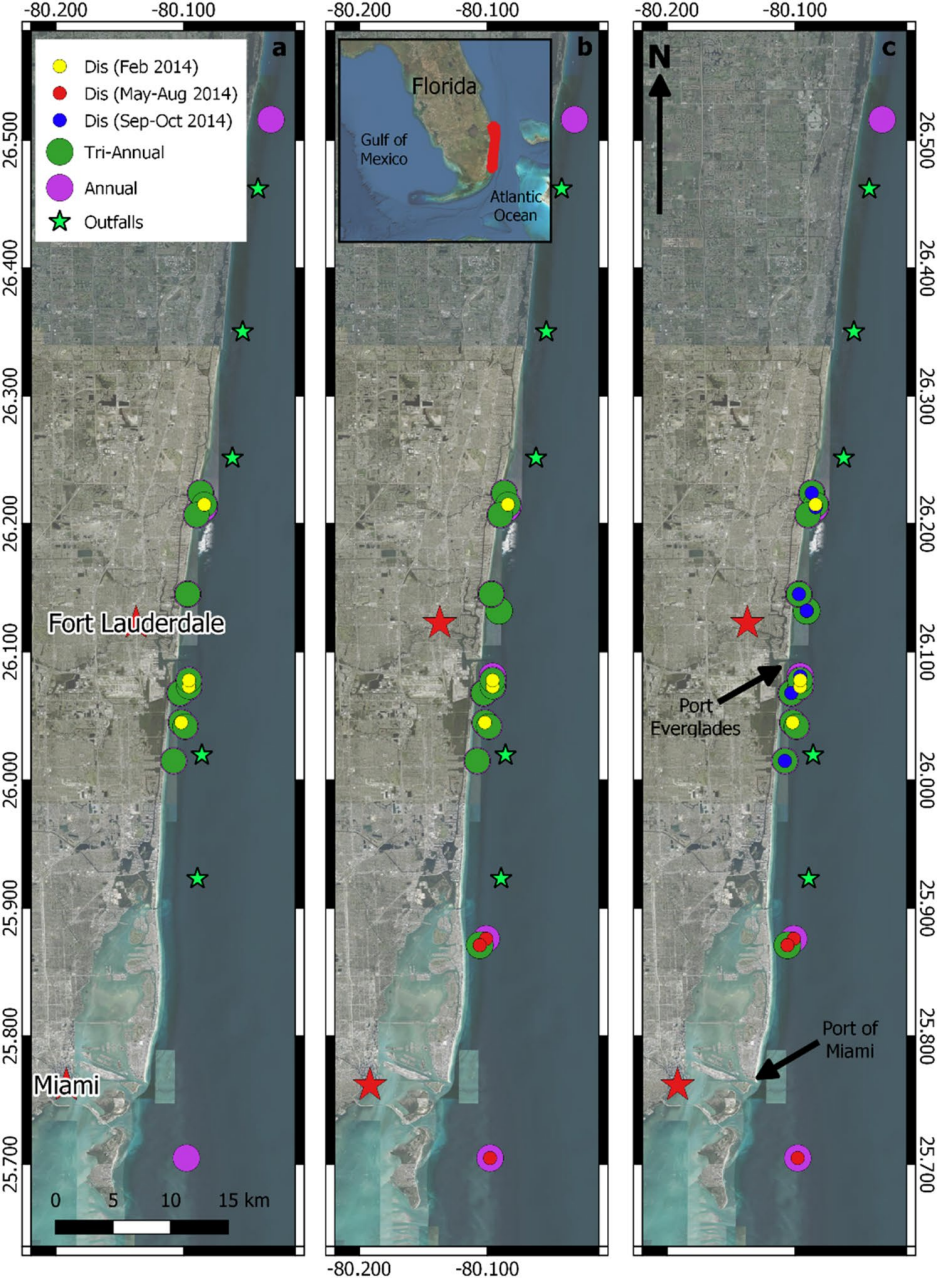
*Dendrogyra cylindrus* colony locations and first incidence of disease (Dis) in the ECA at three timepoints, (**a**) Winter 2014 (Feb), (**b**) Summer 2014 (May–Aug), (**c**) Fall 2014 (Sep–Oct). Inset middle = Florida Peninsula with ECA highlighted in red. No colony suffered 100% mortality during this period. Figure created using QGIS software 3.18.12.

No clear spatial pattern in disease incidence, prevalence or severity was found and no leading edge of disease regional progression was identified during the study (Fig. 3). Disease-related mortality was recorded on four of 21 *D. cylindrus* colonies during the first monitoring period in February 2014. These colonies were located 14 km north and within 6 km south of Port Everglades. These data are demonstrative of the overall spatial extent of disease as the sites span the full sampling range. Two of six *D. cylindrus* colonies located further south, approximately 12 km north of Port of Miami, had disease-related mortality when they were first monitored in May 2014. Disease-related mortality on the southernmost monitored colony, located offshore Virginia Key in Miami-Dade County, was not recorded until August 2014. By October 2014, disease was recorded at 14 of 24 sites. Disease was first recorded on one of two colonies at the northernmost annual site in Palm Beach County in July 2015, with both colonies dying in April 2016. Whole colony mortality was first recorded in September 2015 (6 of 34 colonies, located 3–25 km south of Port Everglades) and all monitored colonies had either experienced complete mortality or had active disease lesions by April 2016.

### Mortality rate

**Figure 4:**
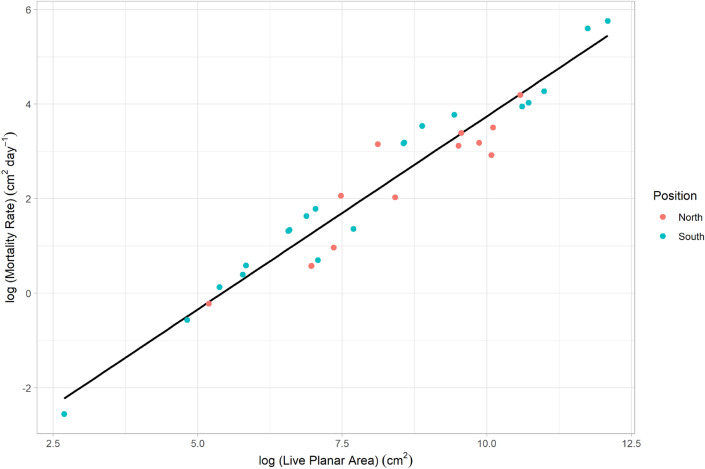
log(Mortality rate) (cm^2^ day^−1^) vs log(colony size) (live planar area cm^2^). Position denotes north or south of Port Everglades. Linear equation: Mortality rate = 4.65 + 0.844 × log(Live Planar Area (cm^2^)).

Mean live tissue loss rate (mortality rate from disease) was 35.3 cm^2^ day^−1^ ± 70.1 cm^2^ (1 SD), equating to 4.7% ± 0.7 (SE) of live tissue lost per month (Fig. 4). A significant positive relationship was found between mortality rate and colony live planar tissue area (Linear regression; p = 0.01, R^2^ = 0.93, n = 33). Colony location, north or south of Port Everglades did not significantly affect mortality rate (t-test, p > 0.05, n = 33). Survival duration was significantly affected by colony size (Survival analysis, z = 3.538, p = 0.0004, n = 33; Supplementary Fig. 1), with larger colonies having a higher survival probability over time than smaller colonies (z = 2.989, p = 0.0028, n = 33). Site depth and colony growth form did not affect survival duration (Survival analysis, p > 0.05).

## Discussion

Here, we document the loss of 66 known, pillar coral (*D. cylindrus*) colonies in the northern portion of Florida’s Coral Reef, the Southeast Florida Coral Reef Ecosystem Conservation Area (ECA), and therefore its likely local ecological extinction. One of seven Caribbean coral species listed as Threatened under the US Endangered Species Act^44^, *D. cylindrus* is rare throughout its range^24,25^ and many populations have been reduced to the point that they are now likely to be reproductively extinct making recovery highly unlikely^32^. Rare coral species have been previously described as having low functional redundancy within coral reef ecosystems, with their loss disproportionally reducing ecosystem function^11^. The loss of *D. cylindrus* represents another case of coral decline in southeast Florida, where macroalgae cover has increased in relation to coral cover loss in recent years^23^. Community change has been widespread in the ECA in recent years^23,39^ and the local extinction of *D. cylindrus* provides further evidence of low stony coral resilience in many species to recent environmental stressors.

Our study contributes to the discussion into the timing, emergence and spread of a disease outbreak attributed to Stony Coral Tissue Loss Disease (SCTLD) in Florida. While we cannot confirm the disease we report is SCTLD, it remains unclear whether SCTLD remains a single disease or has a single pathogen^40^, *D. cylindrus* is considered highly susceptible to SCTLD^41^, many disease lesions and progressions observed here superficially resemble those described for SCTLD (multifocal white lesions often originating within live tissue) and the timing of its loss was at the onset of the disease. During our study, white-syndrome type disease lesions were first recorded in ECA *D. cylindrus* colonies in February 2014 on four colonies (19% prevalence) located north and south of Port Everglades. Whole colony mortality was first recorded in September 2015, and by April 2016, 87% of monitored colonies were recorded dead. Unlike the commonly accepted narrative implying that SCTLD originated offshore Virginia Key, spreading north towards Martin County and south to the Florida Keys^41,42^, no clear spatial pattern in disease spread or mortality was found here. No correlation between disease prevalence and colony location was found, with disease occurrences found north and south of Port Everglades in the two monitoring periods following the first record of disease. We suggest that for *D. cylindrus* at least, the disease outbreak did not have a single point of origin or initial linear spread, but that thermal anomalies, consecutive bleaching years, and other environmental conditions facilitated its outbreak in the ECA, before spreading south to the Florida Keys.

**Figure 5:**
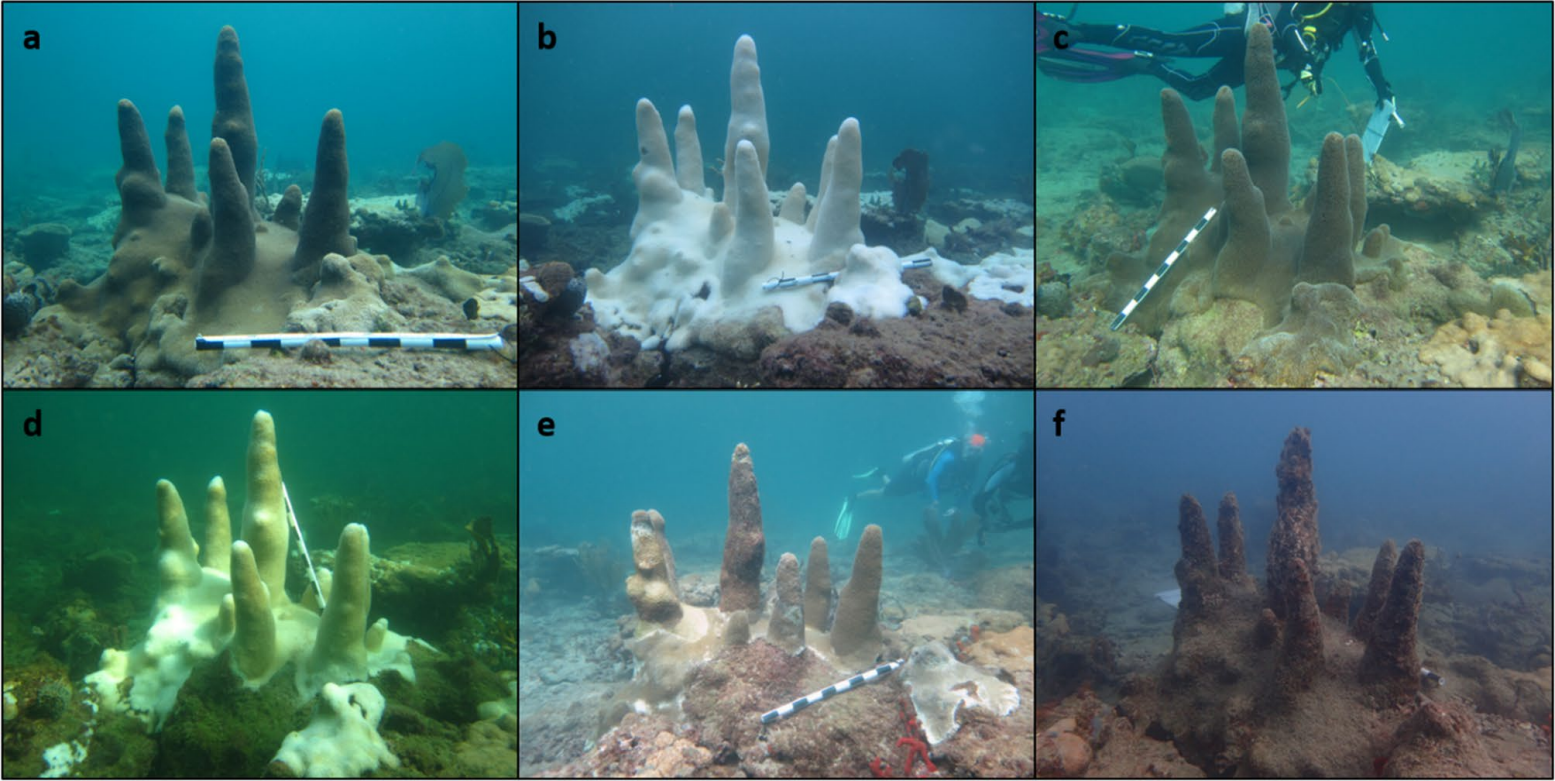
*Dendrogyra cylindrus* colony L–R from top: (**a**) Spring 2014 (healthy), (**b**) Fall 2014 (bleached), (**c**) Winter 2015 (recovered), (**d**) Fall 2015 (bleached and diseased), (**e**) Fall 2016 (diseased), (**f**) Winter 2018 (dead).

Disease prevalence and severity was positively related to heat stress stemming from consecutive El Niño events in 2014 and 2015, during which 88% and 81% of colonies bleached. Correlations between heat stress, stony coral bleaching and disease are long established^17,45–48^ and a positive relationship between stony coral cover decline and heat stress duration was identified in the ECA^23^. *Dendrogyra cylindrus* colonies bleached in fall 2014, recovered during winter and then re-bleached in fall 2015 (Fig. 5). Disease-related recent mortality followed a similar pattern, as it did during a black band disease outbreak in the Florida Keys^49^. Disease prevalence was positively related to the maximum temperature between monitoring periods and disease severity was positively related to mean temperature between monitoring periods. In situ water temperature peaked in summer 2014, causing widespread bleaching of *D. cylindrus* colonies and facilitated an increase in disease prevalence. Mean temperature between monitoring periods peaked in summer 2015, further stressing *D. cylindrus* colonies and causing prolonged bleaching and increasing disease severity, ultimately killing 87% of colonies by early 2016. Our findings support other studies that conclude repeat bleaching events reduce coral fitness, promote disease outbreaks and can lead to mass mortality events^15^. The near local extinction of *D. cylindrus* adds to a growing body of evidence suggesting that the increasing frequency and severity of bleaching events under climate change greatly threatens the long-term survival of coral reefs^15,16^.

Thermal stress likely exacerbated disease conditions already present in the ECA, leading to its outbreak^40^, suggesting local stress reduced resilience, contributing to the emergence of SCTLD. Sources of potential chronic stress related to water quality include ports and wastewater outfalls. The first diseased colonies at our sites were located near Port Everglades, which brings elevated nutrients and brackish water to the surrounding environment. Three of these colonies were also inshore of Hollywood outfall, which discharges partially treated wastewater four kilometers offshore^36^. Local environmental stressors, such as light attenuation^50,51^, sedimentation^21^ and nutrient enrichment^19,52^ have all been linked to reduced resilience and/or stony coral disease and are all prevalent in the ECA. Regardless of the exact causative agent, our findings contribute to increasing evidence that environmental conditions and a changing community composition in the ECA are detrimental to stony coral survival and recovery from disturbance^23,39^.

Disease-related mortality of *D. cylindrus* colonies was rapid, with an average of 4.7% live tissue lost per month and mean mortality rate of 35.3 cm^2^ day^−1^. Our mortality rate metric is likely an underestimate as it does not account for colony height, which given the colony growth form, did not allow for accurate calculation of three dimensional size, but it provides a useful comparison to other studies. Aeby et al.^40^ found *Montastraea cavernosa* colonies, which are considered moderately susceptible to SCTLD^41^, lost on average 2.83% of tissue per month (4.2–0.8 cm^2^ day^−1^) from SCTLD. Lewis et al.^49^ found black band disease progressed at 0.5 cm day^−1^ on *D. cylindrus* in the Florida Keys. Voss and Richardson^53^ found black band disease progressed 0.6 mm day^−1^ on *Siderastrea siderea* colonies under ambient in situ conditions, with progression increasing under elevated nutrient concentrations, as nutrient concentrations are in the ECA. Our findings and these comparisons corroborate reports that this white-syndrome type disease is particularly virulent in *D. cylindrus*^41^. Mortality rate and survival probability declined with colony size, but was independent of colony type, depth or location. While tissue loss was slower on small colonies, their size still hindered survival probability, with the reverse for large colonies. Any future management effort with limited resources may consider focusing rapid response treatments to large colonies at the first sign of disease.

During our 6-year study we documented the ecological extirpation of the entire ECA *D. cylindrus* population. A study initially developed to assess the status of a rare, threatened species at the northern limit of its range captured the loss of all 66 monitored colonies in just a few years. Our findings suggest that a white-syndrome type disease was present above outbreak levels (> 5% as defined for the coral population by Precht et al.^42^) on *D. cylindrus* as early as February 2014 and disease severity was greatly exacerbated by heat stress from two consecutive bleaching events in 2014 and 2015. Disease prevalence was strongly correlated with high maximum water temperature and prolonged in situ water temperature above the bleaching threshold in 2015 and culminated in whole colony mortality of 87% of the ECA *D. cylindrus* population between September 2015 and January 2016. By March 2018, no live tri-annually monitored colonies remained, with the final monitored colony dying in June 2020. The extinction of *D. cylindrus* in southeast Florida presents a precedent for other threatened species and the dangers faced by the dual threat of increasing anthropogenically induced local stress and climate change^14,16^.

## Methods

### Data collection

Between 2013 and 2014, the locations of all known *D. cylindrus* colonies identified by the scientific and lay community on Florida’s Coral Reef were collated in a study coordinated by the Florida Fish and Wildlife Conservation Commission (FWC). In the ECA, ground truthing of these locations was conducted from October 2013 to April 2014 and 27 colonies were confirmed. Between April 2014 and December 2016, 39 additional colonies were identified and the status of these 66 colonies is described herein. Thirty-four *D. cylindrus* colonies from 12 sites were monitored tri-annually from 2014 to 2018 to determine the status of the pillar coral population in the ECA (hereafter referred to as tri-annual colonies). Ten additional tri-annual colonies were monitored from 2015 to 2017 but were not used in statistical analyses. Twenty-two colonies from 12 sites, representing all other known *D. cylindrus* colonies in the ECA were monitored once per year from 2014 to 2018 (hereafter referred to as annual colonies). Tri-annual sites were visited ten times during the project (February 2014, May 2014, September 2014, January 2015, June 2015, September 2015, March/April 2016, September 2016, February 2017 and March 2018). Eighteen tri-annual and three annual colonies were surveyed by the first monitoring event in February 2014 and all 34 tri-annual colonies surveyed from May 2014 until they died. Annual sites were visited in 2014, 2015, 2016, 2017 and 2018, and typically monitored during the summer months between May and September. Statistical analyses and associated figures incorporated the original 34 tri-annual colonies only. Qualitative assessments and disease progression maps include all tri-annual and annual colonies.

*Dendrogyra cylindrus* monitoring protocols were developed through a collaboration between FWC and Nova Southeastern University (NSU) and were modified from the *Acropora palmata* demographic monitoring protocol written by Williams et al.^54^. Colony growth form (encrusting, pillar, encrusting with pillars or attached fragment) and location depth and habitat were recorded for all colonies. During each monitoring period, colony data was collected including colony size (length, width, height), percent live tissue, percent and cause of recent mortality (typically disease or competition with other organisms), and presence and percent of tissue affected by bleaching. Disease was the appearance of a lesion defined by the presence of recent mortality and bleaching was white or near-white live tissue (i.e. no recent mortality). Disease-related mortality was differentiated from additional sources of mortality by assessment of skeletal structure, adjacent tissue margin, rate of spread and presence/absence of adjacent organisms which may cause mortality (e.g. abrasion). Based on gross pathology, white-syndrome type disease was attributed to focal, multifocal or diffuse tissue loss with no skeletal damage, no abnormal skeletal or tissue growth, lesion boundaries free of adjacent pigmented mats or band, no signs of organismal abrasion or overgrowth^55–57^. Additionally, during most events top down and side view colony images were taken to reference conditions noted during data collection and for comparison to preceding monitoring periods. Temperature data was collected bi-hourly using HOBO Pro v2 temperature loggers located at six nearby Southeast Florida Coral Reef Evaluation and Monitoring Project (SECREMP) sites^58^. Data from these six sites were chosen based on proximity and comparable depths to *D. cylindrus* monitoring locations. This study was conducted in compliance with the ARRIVE guidelines, methods were carried out in accordance with Florida Fish and Wildlife Conservation Commission Program (FWC) regulations and monitoring protocols approved by FWC under Special Activity License SAL-13-1451-SRP.

### Data analysis

*Dendrogyra cylindrus* disease and mortality dynamics were assessed in relation to population demographics (colony size and growth form), spatial distribution (latitude and depth) and in situ water temperature. Statistical data analyses were conducted in R^59^. Colony size was assessed as planar live tissue area (LTA; cm^2^). LTA was calculated by multiplying colony planar area (maximum diameter × perpendicular width), by percent live tissue estimated during in situ assessment as per Walton et al.^39^ and Aeby et al.^40^. Two-dimensional LTA was calculated as *D. cylindrus* colony height is not uniform, due to the presence of pillars, making three-dimensional LTA less accurate. LTA was calculated for each colony during each monitoring period. A semi-quantitative estimate of mortality rate was calculated by dividing the change in colony LTA from the first recorded sign of disease (Initial LTA) to the final monitoring period or to colony death (End LTA) by time (Days; Eq. 1). Only tri-annually monitored colonies where disease presence was captured prior to death were included in statistical analyses. Mortality rate was statistically assessed using model 2 regression in relation to LTA and using a t-test in relation to colony location, north or south of Port Everglades. Data were log transformed for normality.

1 $${\text{Mortality}}\;{\text{Rate}} = {\text{Initial}}\;{\text{LTA}}{-}{\text{end}}\;{\text{LTA/Time}}$$

A survival analysis was conducted to assess whether survival duration varied by site depth, colony size, or colony growth form, using a Cox model. To assess size, colonies were grouped by planar LTA into size classes: small (< 1000 cm^2^), medium (1000–9999 cm^2^), large (10,000–49,999 cm^2^) and extra-large (> 50,000 cm^2^).

The relationship between temperature and disease was assessed in two forms. Maximum, mean and minimum temperatures between monitoring periods were calculated for each site using data from in situ bi-hourly temperature loggers placed at nearby long-term monitoring sites^23^. A stepwise Generalized Linear Model (GLM), with a quasi-binomial distribution was used to assess the relationship between disease prevalence (percentage of diseased colonies per monitoring period) and maximum, mean and minimum temperature. A Chi-squared ANOVA was used to assess the significance of variables in the minimum adequate model. Model validation was assessed by plotting deviance residuals and fitted values, and deviance residuals against each significant variable in the minimum adequate model. A Generalized Linear Mixed Model (GLMM) was fitted to assess the relationship between percent disease-related recent mortality and bleaching prevalence, maximum temperature, mean temperature and minimum temperature, but model fit was unreliable. A Spearman’s rank correlation was instead conducted to assess the relationship between mean temperature (the only variable in the tested minimum adequate GLMM) and percent disease-related recent mortality (recent mortality on each individual colony in each monitoring period).

## Supplementary Information

Supplementary Information.

## Data Availability

Data that supports the findings of this study are available from the corresponding author on reasonable request.
