# Supplementary material for: Temperature stress and disease drives the extirpation of the threatened pillar coral, Dendrogyra cylindrus, in southeast Florida
Source: Sci Rep. 2021 Jul 8;11:14113. doi: 10.1038/s41598-021-93111-0 (PMC8266880; doi:10.1038/s41598-021-93111-0)
Supplement: Supplementary file 1 — Supplementary Information. [file 41598_2021_93111_MOESM1_ESM.docx]

**Temperature stress and disease drives the extirpation of the threatened pillar coral, *Dendrogyra cylindrus,* in southeast Florida**

Nicholas P. Jones^1*+^; Lystina Kabay ^1+^; Kathleen Semon Lunz^2^; David S. Gilliam^1^

^1^Nova Southeastern University, Halmos College of Arts and Sciences, Dania Beach, FL, USA

^2^National Operations Center, Bureau of Land Management, Denver, CO, USA

^*^Corresponding Author

^+^Co First Authors

**Supplementary Materials**


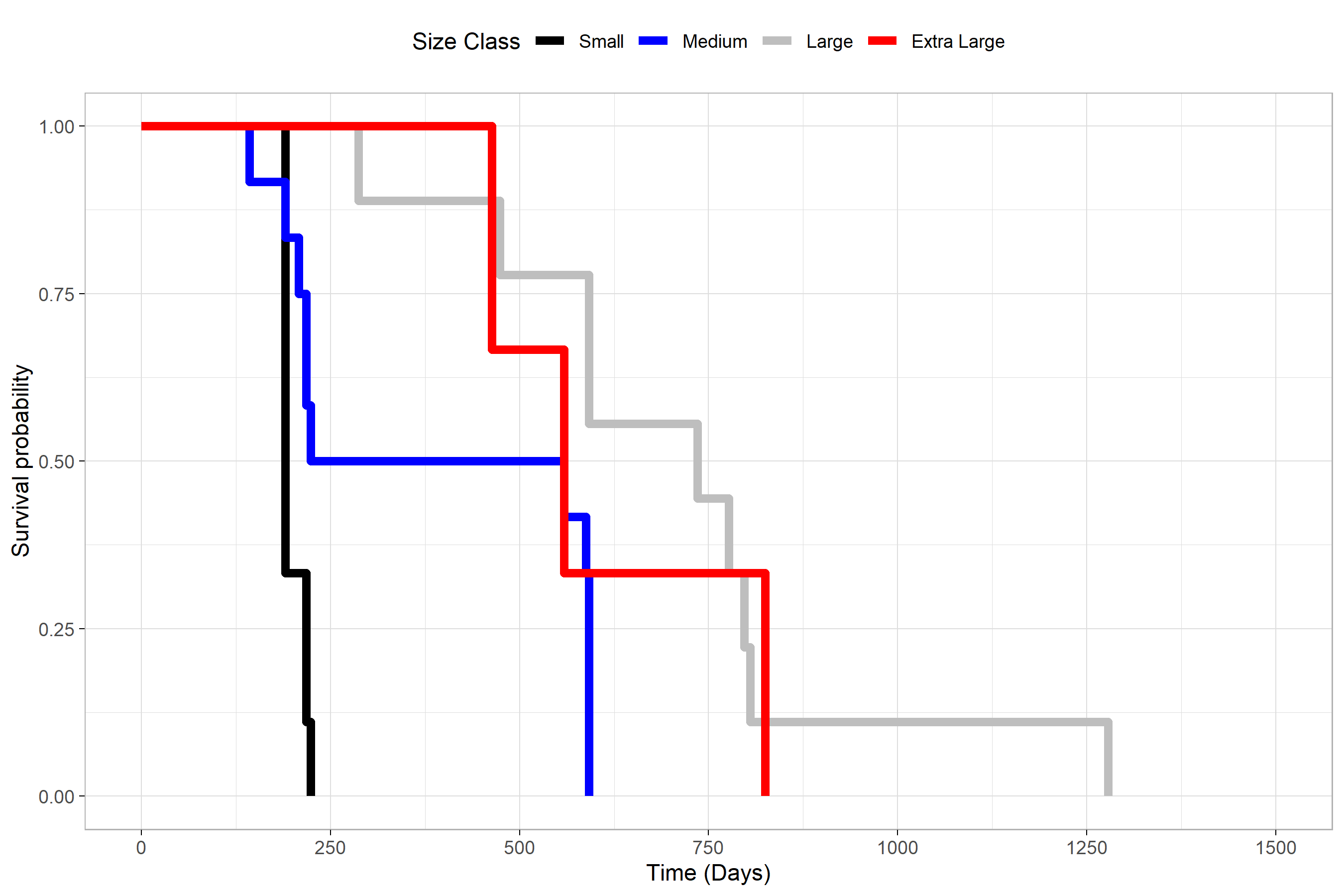


Supplementary Figure 1. Survival Analysis (Cox Model) assessing survival probability over time, by colony size class.


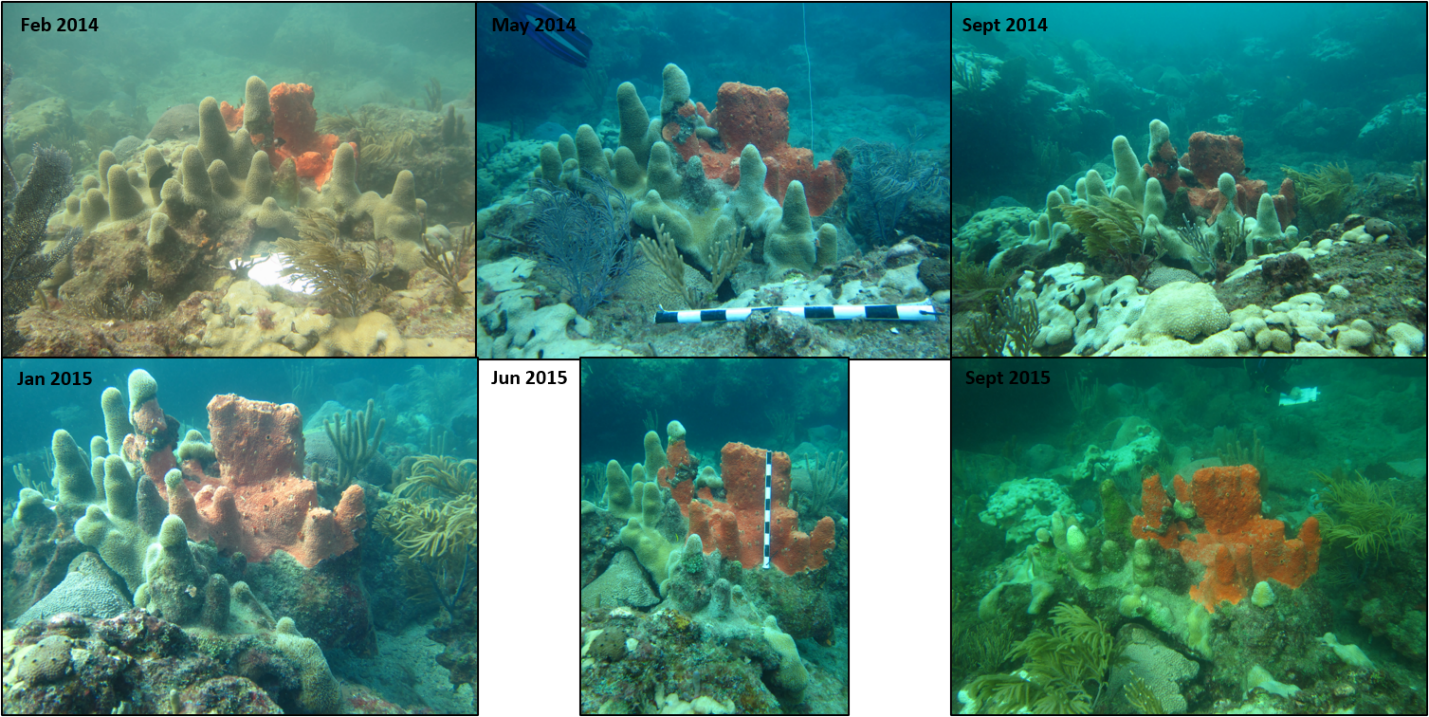


Supplementary Figure 2. Dendrogyra cylindrus colony DAP8 from Winter 2014 to Fall 2015.


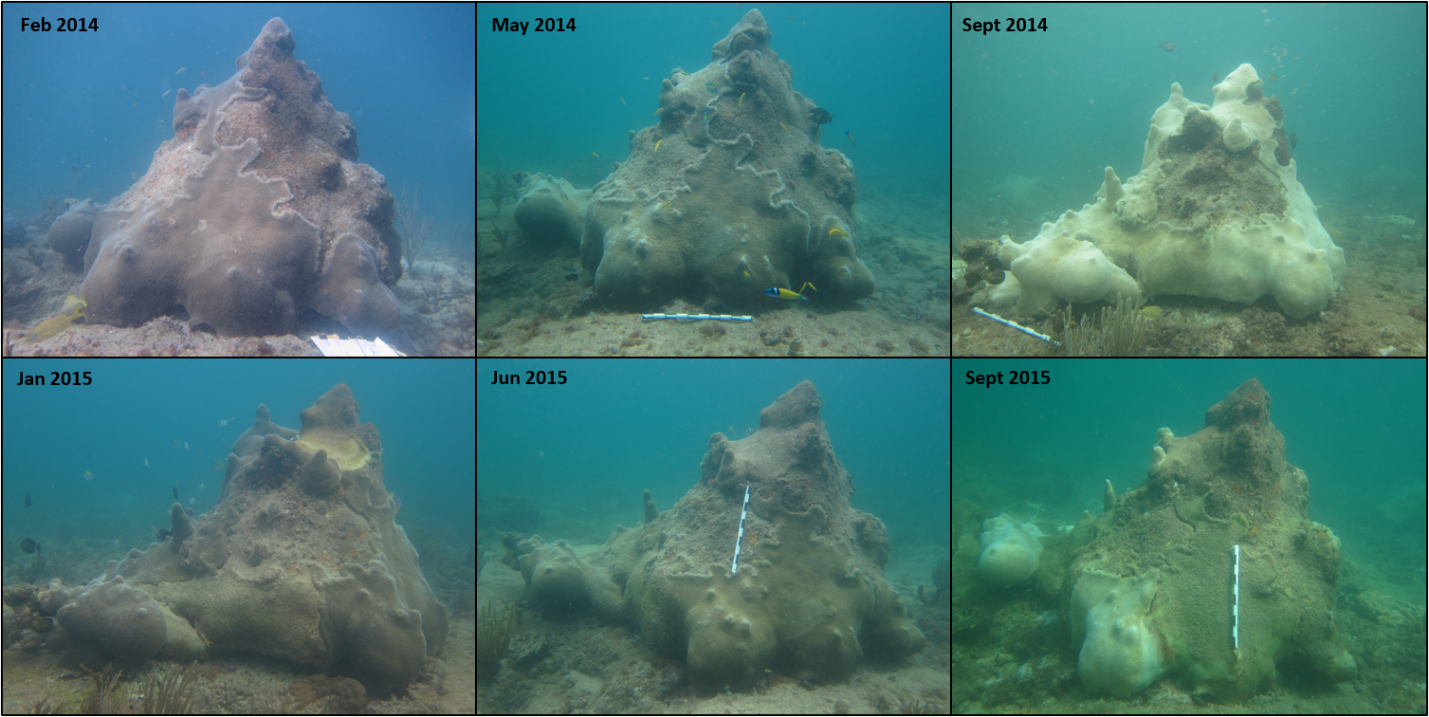


Supplementary Figure 3. Dendrogyra cylindrus colony DAP10 from Winter 2014 to Fall 2015. Note bleaching in September 2014 and September 2015 and disease in January 2015.


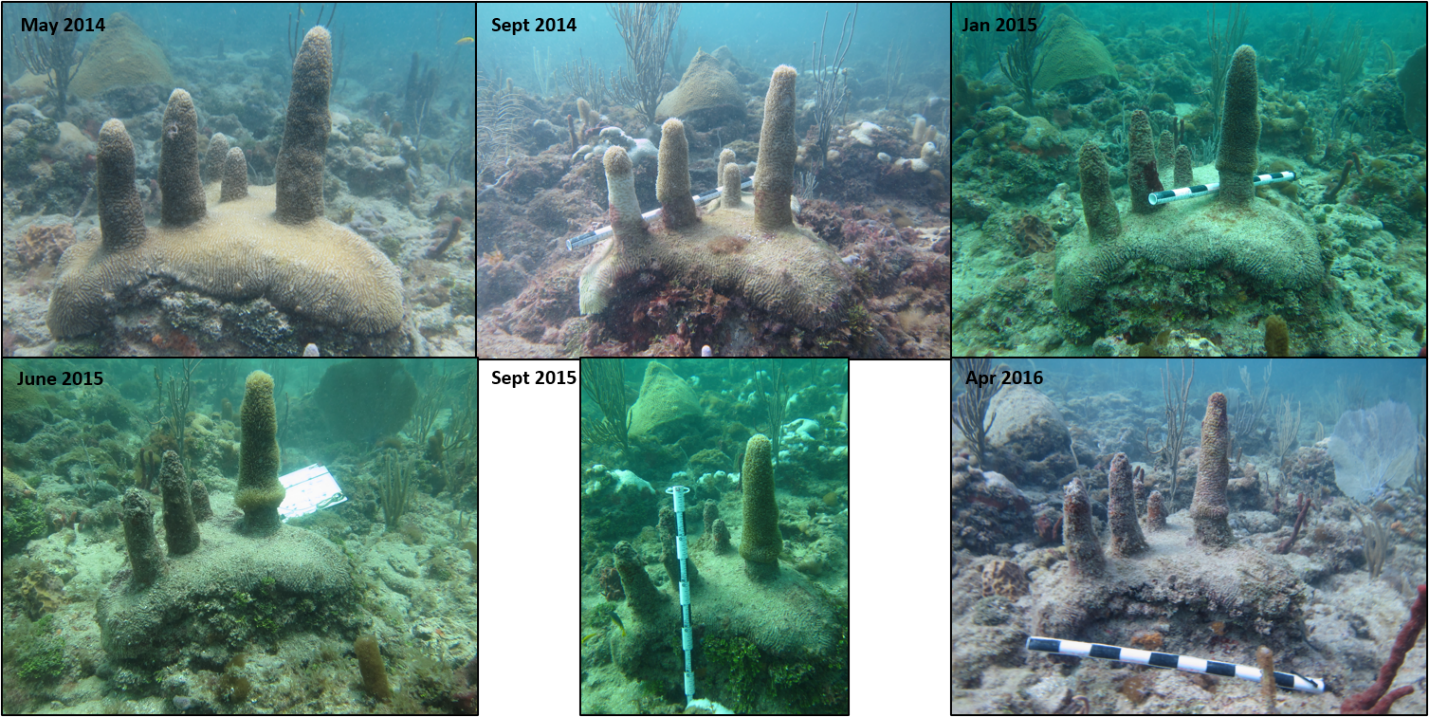


Supplementary Figure 4. Dendrogyra cylindrus colony DAP13 from Spring 2014 to Spring 2016. Note extensive disease in September 2014.


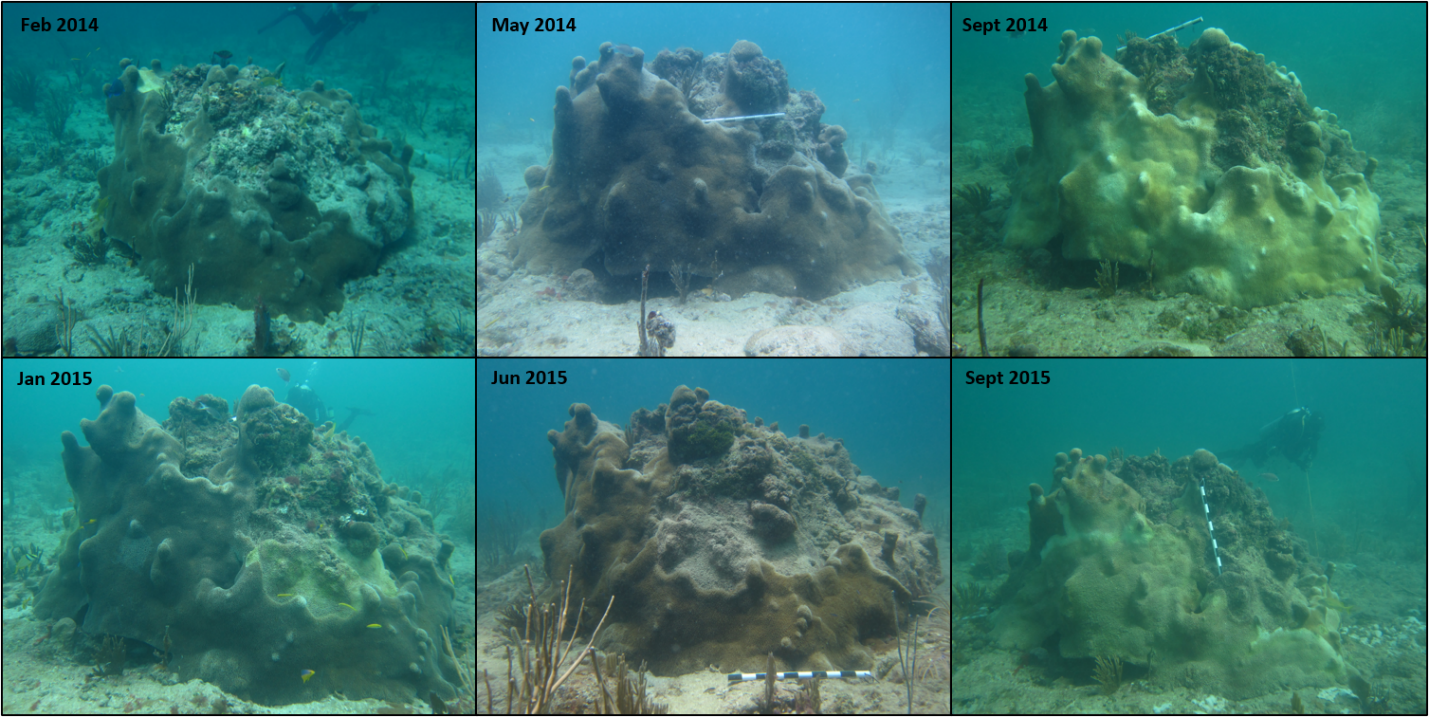


Supplementary Figure 5. Dendrogyra cylindrus colony DAP14 from Spring 2014 to Fall 2015. Note disease on upper left of colony in February 2014 (Top left).


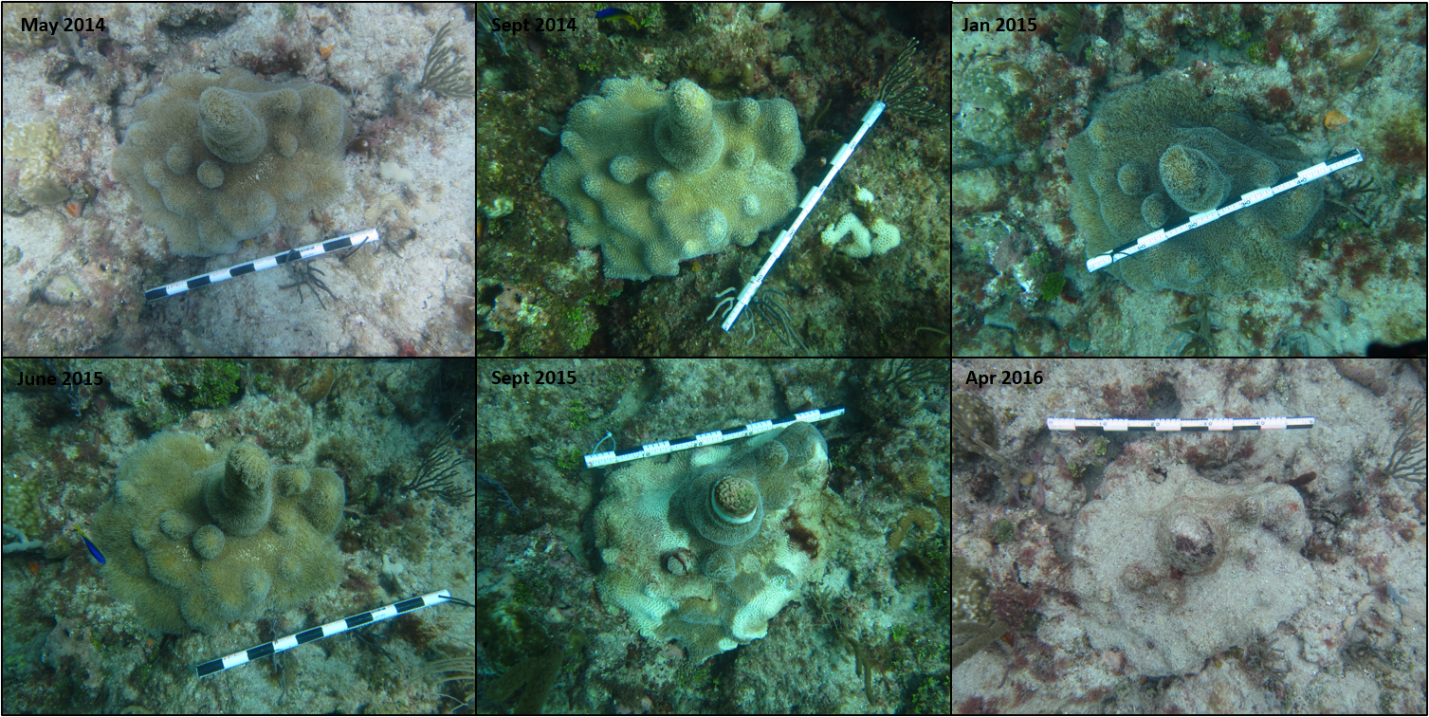


Supplementary Figure 6. Dendrogyra cylindrus colony DAP15B from Spring 2014 to Spring 2016. Note paling in September 2014, extensive disease related mortality in September 2015.


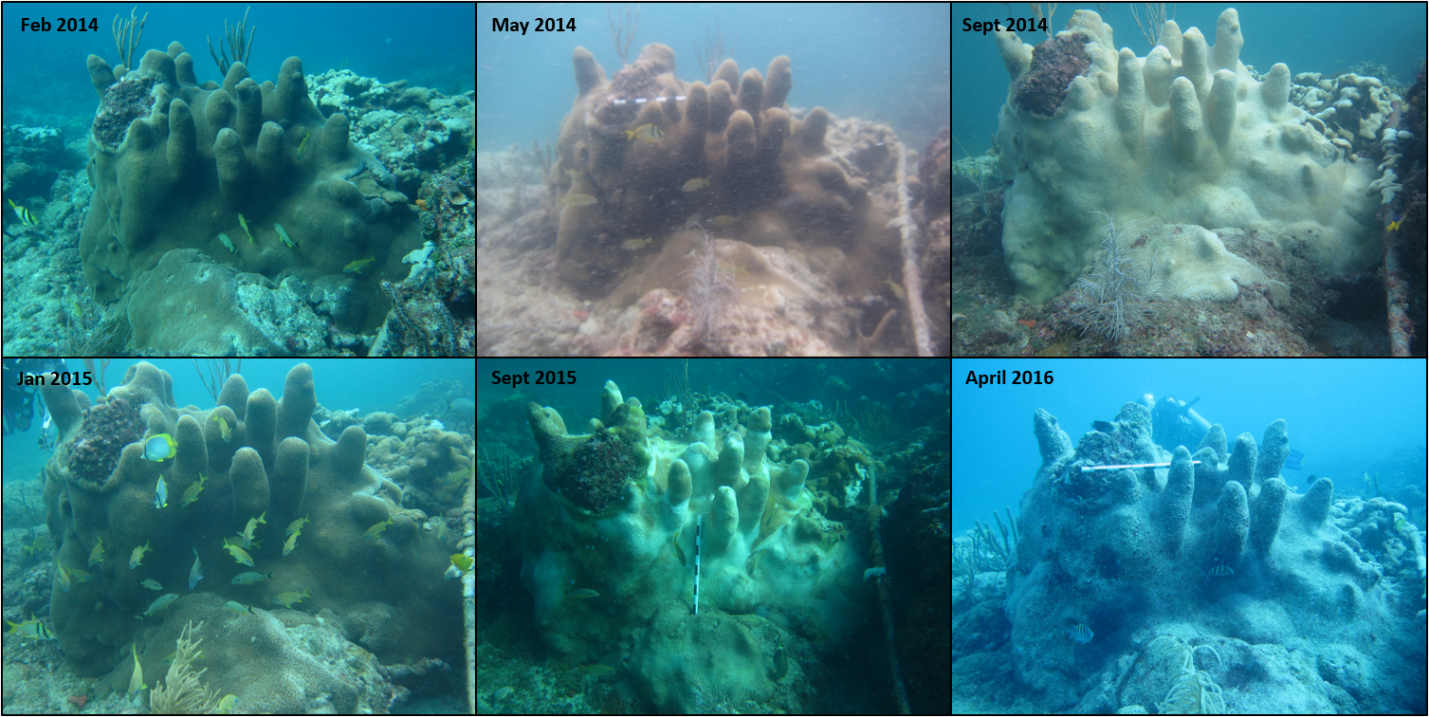


Supplementary Figure 7. Dendrogyra cylindrus colony DAP19A from Winter 2014 to Spring 2016. Note bleaching in September 2014, bleaching and disease September 2015.


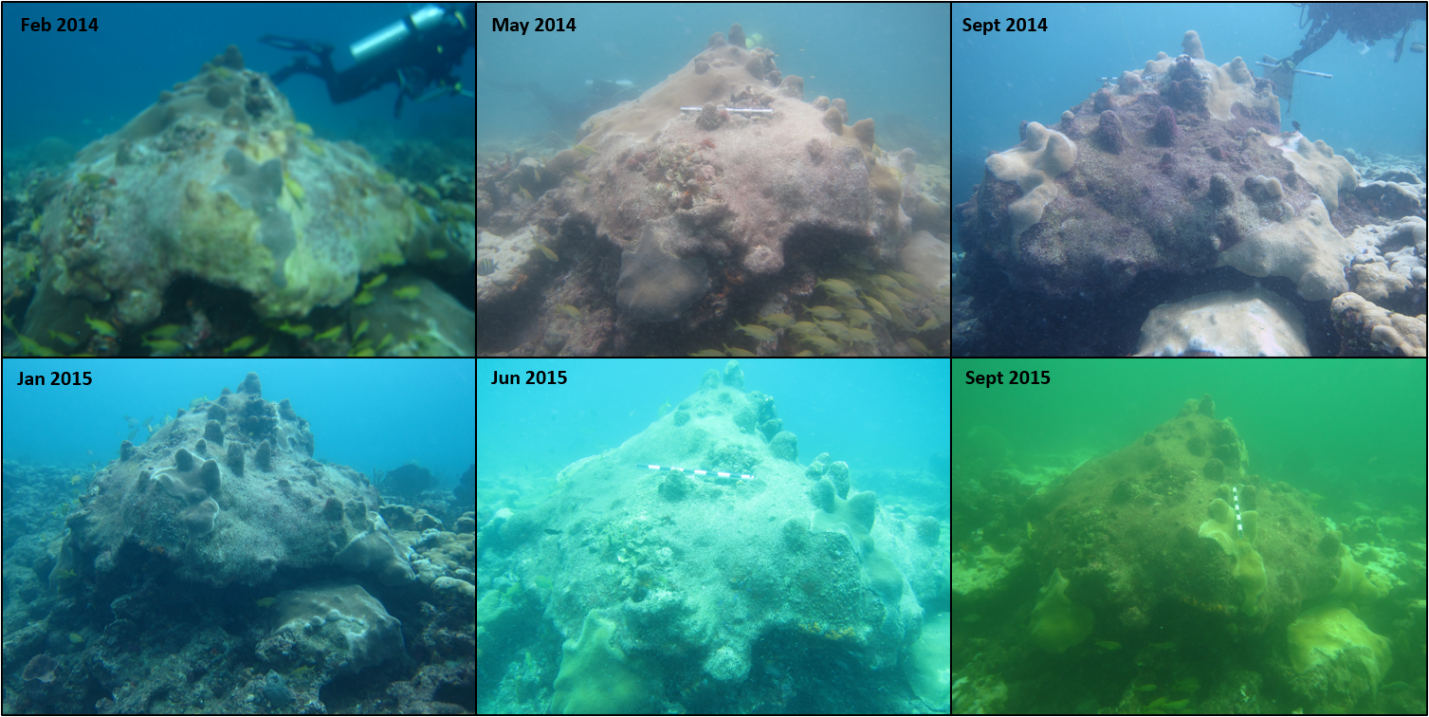


Supplementary Figure 8. Dendrogyra cylindrus colony DAP20 from Winter 2014 to Fall 2015. Note disease February 2014 (center of colony).


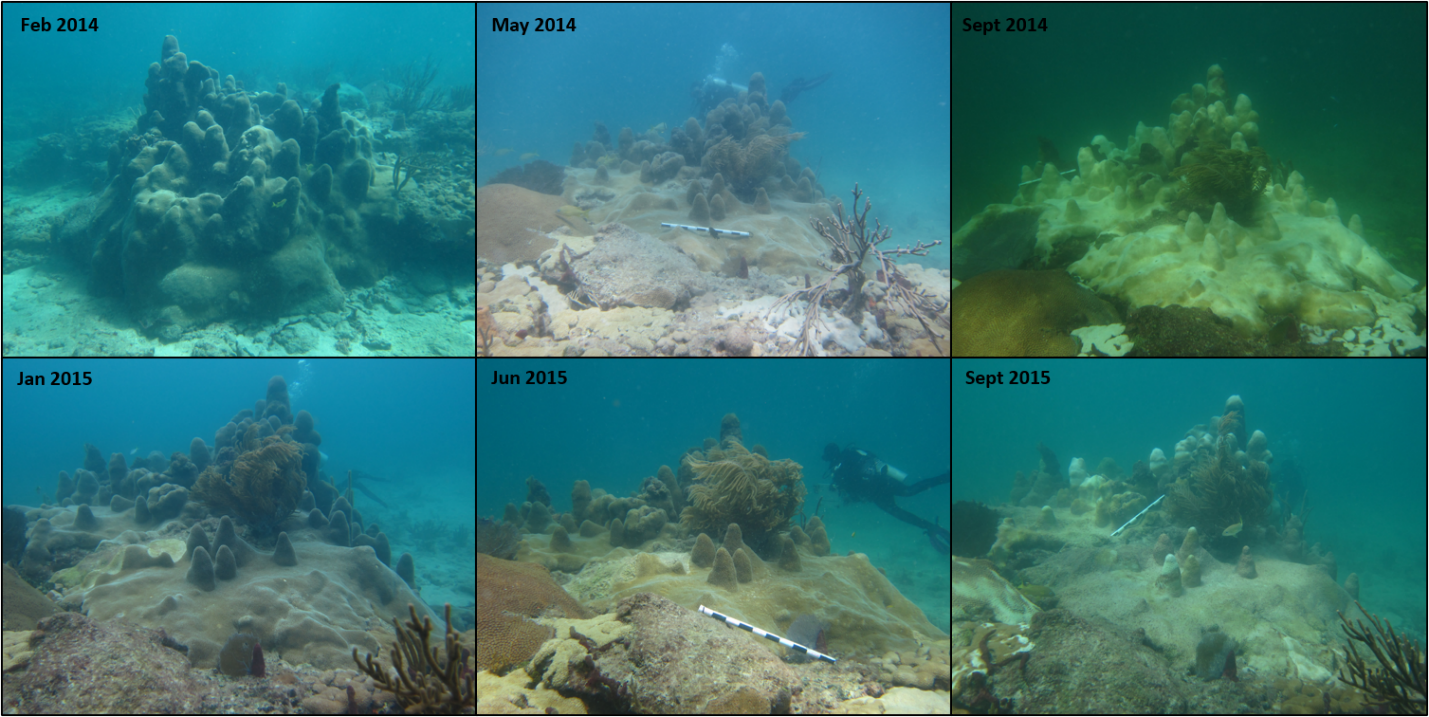


Supplementary Figure 9. Dendrogyra cylindrus colony DAP21A from Winter 2014 to Fall 2015. Note bleaching in September 2014 and disease on left of colony in January 2015.


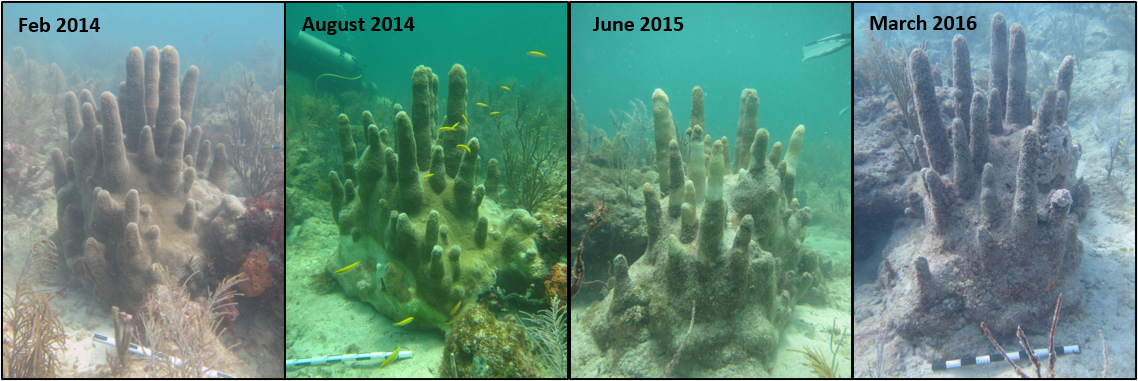


Supplementary Figure 10. Dendrogyra cylindrus colony DAP23 from Winter 2014 to Winter 2016.


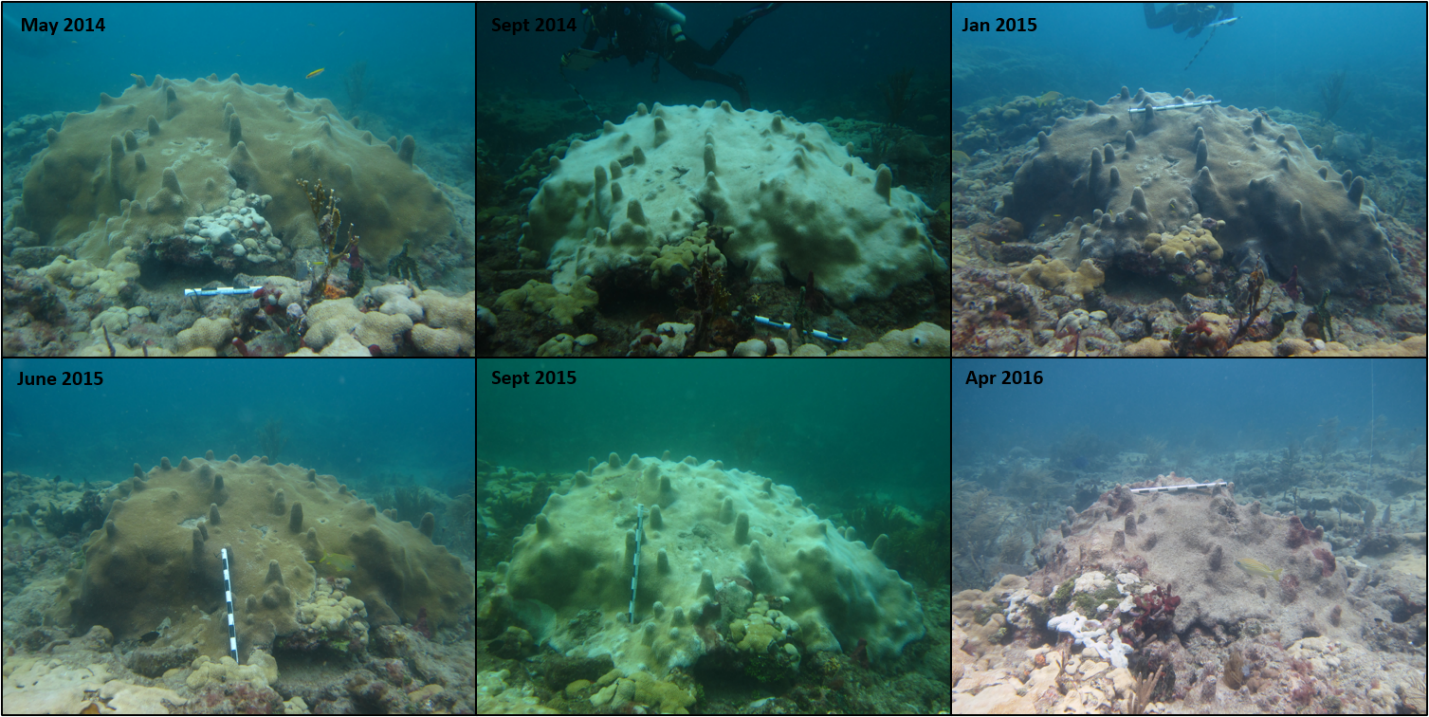


Supplementary Figure 11. Dendrogyra cylindrus colony DAP29A from Spring 2014 to Spring 2016. Note bleaching September 2014 and September 2015.


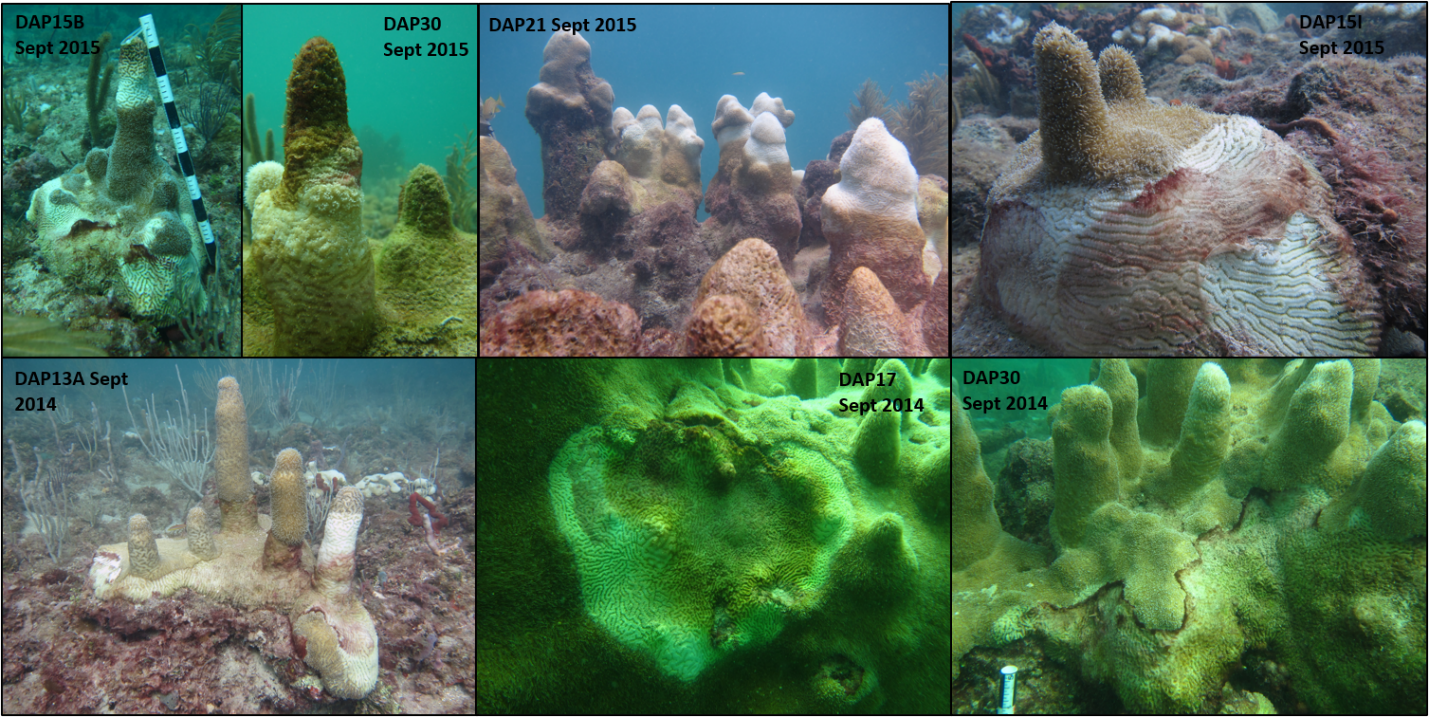


Supplementary Figure 12. Disease lesions.


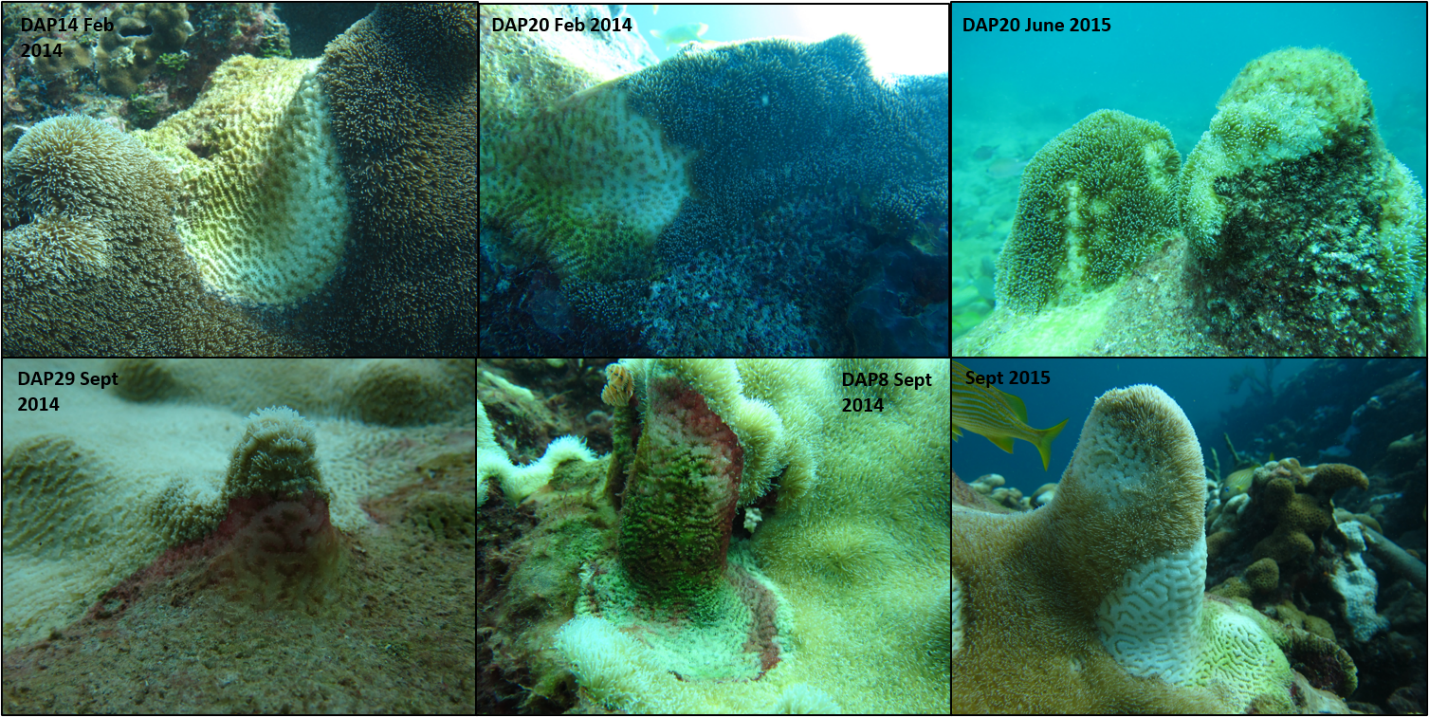


Supplementary Figure 13. Disease lesions. Note disease lesions in February 2014 (Upper left and upper middle).

Supplementary Table 1. Colony Information. Location = county colony located in; Broward north/south refers to position in relation to Port Everglades in Broward County. Colony type: ENPI = Encrusting with Pillars; PI = Pillar; EN = Encrusting; AF = Attached fragment. Date first disease = Date disease-related mortality first observed on colony.

| Colony ID | Monitoring Frequency | Location | Depth (ft) | Colony Type | Length (cm) | Width (cm) | Height (cm) | Date First Disease | Date 100% Mortality |
| --- | --- | --- | --- | --- | --- | --- | --- | --- | --- |
| DAP01-A | Annual | Palm Beach | 53 | ENPI | 58 | 38 | 20 | 7/13/2015 | 4/14/2016 |
| DAP02-A | Annual | Palm Beach | 64 | ENPI | 315 | 282 | 69 | None seen | 4/14/2016 |
| DAP07-A | Tri-annual | Broward – north | 15 | PI | 173 | 153 | 115 | 9/11/2014 | 3/12/2018 |
| DAP08-A | Tri-annual | Broward – north | 13 | PI | 192 | 143 | 97 | 2/7/2014 | 4/14/2016 |
| DAP09 | Tri-annual | Broward – north | 14 | ENPI | 69 | 57 | 50 | 4/25/2016 | 9/15/2016 |
| DAP10 | Tri-annual | Broward – north | 15 | ENPI | 220 | 185 | 170 | 9/11/2014 | 9/15/2016 |
| DAP10-AF1 | Tri-annual | Broward – north | 15 | AF | 45 | 40 | 25 | 9/14/2015 | 4/25/2016 |
| DAP13-A | Tri-annual | Broward – south | 22 | PI | 56 | 53 | 54 | 9/16/2014 | 4/26/2016 |
| DAP13-B | Tri-annual | Broward – south | 22 | ENPI | 103 | 63 | 76 | 9/21/2015 | 4/26/2016 |
| DAP13-HC1 | Tri-annual | Broward – south | 22 | ENPI | 38 | 34 | 32 | 9/21/2015 | 4/26/2016 |
| DAP13-HC2 | Tri-annual | Broward – south | 22 | ENPI | 79 | 76 | 70 | 9/21/2015 | 4/26/2016 |
| DAP13-HC3 | Tri-annual | Broward – south | 22 | ENPI | 33 | 31 | 34 | none seen | 4/26/2016 |
| DAP14 | Tri-annual | Broward - south | 21 | ENPI | 246 | 235 | 141 | 2/11/2014 | 3/29/2016 |
| DAP15-A | Tri-annual | Broward - south | 22 | ENPI | 83 | 44 | 36 | none seen | 9/21/2015 |
| DAP15-B | Tri-annual | Broward - south | 22 | ENPI | 48 | 45 | 50 | 9/21/2015 | 3/29/2016 |
| DAP15-C | Tri-annual | Broward - south | 22 | ENPI | 61 | 28 | 34 | 9/21/2015 | 3/29/2016 |
| DAP15-D | Tri-annual | Broward - south | 22 | ENPI | 82 | 59 | 39 | 9/21/2015 | 3/29/2016 |
| DAP15-E | Tri-annual | Broward - south | 22 | ENPI | 145 | 98 | 107 | 9/21/2015 | 3/29/2016 |
| DAP15-F | Tri-annual | Broward - south | 22 | ENPI | 116 | 49 | 79 | 9/21/2015 | 3/29/2016 |
| DAP15-G | Tri-annual | Broward - south | 22 | AF | 86 | 48 | 36 | none seen | 9/21/2015 |
| DAP15-H | Tri-annual | Broward - south | 22 | ENPI | 58 | 41 | 30 | none seen | 9/21/2015 |
| DAP15-I | Tri-annual | Broward - south | 22 | ENPI | 32 | 23 | 20 | 9/21/2015 | 3/29/2016 |
| DAP15-J | Tri-annual | Broward - south | 22 | AF | 17 | 13 | 9 | 9/21/2015 | 9/21/2015 |
| DAP17 | Annual | Broward - south | 24 | ENPI | 393 | 294 | 198 | 10/29/2014 | 4/25/2016 |
| DAP19-A | Tri-annual | Broward - south | 28 | ENPI | 266 | 200 | 144 | 2/11/2014 | 4/26/2016 |
| DAP19-AF1 | Tri-annual | Broward - south | 28 | AF | 26 | 17 | 10 | none seen | 9/21/2015 |
| DAP19-AF2 | Tri-annual | Broward - south | 28 | AF | 15 | 11 | 4 | 9/21/2015 | 4/26/2016 |
| DAP20 | Tri-annual | Broward - south | 20 | ENPI | 335 | 295 | 165 | 2/11/2014 | 5/16/2016 |
| DAP21-A | Tri-annual | Broward - north | 20 | ENPI | 569 | 446 | 236 | 9/17/2014 | 3/29/2016 |
| DAP23 | Annual | Miami-Dade | 21 | PI | 186 | 138 | 115 | 8/7/2014 | 3/29/2016 |
| DAP25 | Annual | Broward - north | 15 | PI | 200 | 150 | 200 | 10/29/2014 | 9/15/2016 |
| DAP25-AF1 | Annual | Broward - north | 15 | AF | 45 | 37 | 22 | 10/29/2014 | 9/15/2016 |
| DAP26-A | Annual | Broward - north | 14 | ENPI | 75 | 71 | 35 | 4/14/2016 | 2/16/2017 |
| DAP28-A | Annual | Broward - north | 36 | ENPI | 345 | 265 | 110 | none seen | 4/14/2016 |
| DAP28-B | Annual | Broward - north | 36 | ENPI | 150 | 115 | 90 | none seen | 4/14/2016 |
| DAP28-C | Annual | Broward - north | 36 | ENPI | 148 | 143 | 72 | None seen | 4/14/2016 |
| DAP29-A | Tri-annual | Broward – north | 22 | ENPI | 236 | 185 | 94 | 9/11/2014 | 4/25/2016 |
| DAP29-B | Tri-annual | Broward - north | 22 | ENPI | 129 | 115 | 76 | 1/7/2015 | 4/25/2016 |
| DAP29-C | Tri-annual | Broward - north | 22 | ENPI | 140 | 84 | 48 | 9/14/2015 | 4/25/2016 |
| DAP29-Dc | Tri-annual | Broward - north | 22 | EN | 108 | 96 | 66 | 9/11/2014 | 4/25/2016 |
| DAP29-E | Tri-annual | Broward - north | 22 | ENPI | 34 | 28 | 30 | None seen | 4/25/2016 |
| DAP29-F | Tri-annual | Broward - north | 22 | ENPI | 57 | 46 | 37 | None seen | 4/25/2016 |
| DAP29-G | Tri-annual | Broward - north | 22 | ENPI | 170 | 113 | 96 | 9/11/2014 | 4/25/2016 |
| DAP29-Hc | Tri-annual | Broward - north | 22 | ENPI | 125 | 120 | 164 | 9/11/2014 | 4/25/2016 |
| DAP29-I | Tri-annual | Broward - north | 22 | ENPI | 40 | 40 | 35 | None seen | 4/25/2016 |
| DAP29-J | Tri-annual | Broward - north | 22 | EN | 32 | 19 | 21 | None seen | 4/25/2016 |
| DAP29-K | Tri-annual | Broward - north | 22 | EN | 20 | 19 | 12 | None seen | 4/25/2016 |
| DAP29-L | Tri-annual | Broward - north | 22 | ENPI | 44 | 30 | 25 | 9/11/2014 | 4/25/2016 |
| DAP29-M | Tri-annual | Broward - north | 22 | ENPI | 40 | 27 | 27 | 9/11/2014 | 4/25/2016 |
| DAP29-N | Tri-annual | Broward - north | 22 | EN | 23 | 14 | 7 | None seen | 4/25/2016 |
| DAP30-A | Tri-annual | Miami-Dade | 18 | ENPI | 139 | 86 | 48 | 9/3/2015 | 3/29/2016 |
| DAP30-B | Tri-annual | Miami-Dade | 18 | PI | 135 | 81 | 59 | 9/17/2014 | 3/29/2016 |
| DAP30-C | Tri-annual | Miami-Dade | 18 | ENPI | 220 | 114 | 98 | 6/16/2015 | 3/29/2016 |
| DAP30-D | Tri-annual | Miami-Dade | 18 | PI | 531 | 394 | 215 | 5/28/2014 | 9/3/2015 |
| DAP31 | Annual | Miami-Dade | 20 | ENPI | 217 | 159 | 70 | 5/28/2014 | 9/15/2016 |
| DAP31-F1 | Annual | Miami-Dade | 20 | AF | 32 | 20 | 7 | None seen | 3/29/2016 |
| DAP32 | Annual | Miami-Dade | 20 | ENPI | 213 | 209 | 67 | 1/20/2015 | 3/29/2016 |
| DAP33-A | Annual | Miami-Dade | 21 | ENPI | 44 | 43 | 41 | None seen | 3/29/2016 |
| DAP33-B | Annual | Miami-Dade | 21 | AF | 23 | 20 | 13 | None seen | 3/29/2016 |
| DAP33-C | Annual | Miami-Dade | 21 | ENPI | 34 | 33 | 29 | None seen | 3/29/2016 |
| DAP33-D | Annual | Miami-Dade | 21 | ENPI | 61 | 51 | 54 | None seen | 5/16/2016 |
| DAP33-E | Annual | Miami-Dade | 21 | ENPI | 79 | 78 | 43 | None seen | 3/29/2016 |
| DAP33-F | Annual | Miami-Dade | 21 | ENPI | 32 | 23 | 15 | None seen | 3/29/2016 |
| DAP34-A | Annual | Broward - north | 27 | ENPI | 290 | 238 | 170 | 11/12/2015 | 2/16/2017 |
| DAP34-B | Annual | Broward - north | 27 | ENPI | 115 | 78 | 45 | 11/12/2015 | 2/16/2017 |
| DAP35 | Observational | Broward - north | 15 | ENPI | 210 | 150 | 105 | 12/22/2016 | 6/30/2020 |
